# Resolving the structure of interactomes with hierarchical agglomerative clustering

**DOI:** 10.1186/1471-2105-12-S1-S44

**Published:** 2011-02-15

**Authors:** Yongjin Park, Joel S Bader

**Affiliations:** 1 Department of Biomedical Engineering, Johns Hopkins University, Baltimore, MD 21218, USA; 2 High-Throughput Biology Center, Johns Hopkins University School of Medicine, Baltimore, MD 21218, USA

## Abstract

**Background:**

Graphs provide a natural framework for visualizing and analyzing networks of many types, including biological networks. Network clustering is a valuable approach for summarizing the structure in large networks, for predicting unobserved interactions, and for predicting functional annotations. Many current clustering algorithms suffer from a common set of limitations: poor resolution of top-level clusters; over-splitting of bottom-level clusters; requirements to pre-define the number of clusters prior to analysis; and an inability to jointly cluster over multiple interaction types.

**Results:**

A new algorithm, Hierarchical Agglomerative Clustering (HAC), is developed for fast clustering of heterogeneous interaction networks. This algorithm uses maximum likelihood to drive the inference of a hierarchical stochastic block model for network structure. Bayesian model selection provides a principled method for collapsing the fine-structure within the smallest groups, and for identifying the top-level groups within a network. Model scores are additive over independent interaction types, providing a direct route for simultaneous analysis of multiple interaction types. In addition to inferring network structure, this algorithm generates link predictions that with cross-validation provide a quantitative assessment of performance for real-world examples.

**Conclusions:**

When applied to genome-scale data sets representing several organisms and interaction types, HAC provides the overall best performance in link prediction when compared with other clustering methods and with model-free graph diffusion kernels. Investigation of performance on genome-scale yeast protein interactions reveals roughly 100 top-level clusters, with a long-tailed distribution of cluster sizes. These are in turn partitioned into 1000 fine-level clusters containing 5 proteins on average, again with a long-tailed size distribution. Top-level clusters correspond to broad biological processes, whereas fine-level clusters correspond to discrete complexes. Surprisingly, link prediction based on joint clustering of physical and genetic interactions performs worse than predictions based on individual data sets, suggesting a lack of synergy in current high-throughput data.

## Background

Graphs or networks provide an excellent organizing framework for representing data from high-throughput experiments that measure interactomes, or genome-scale biological interactions: physical interactions between proteins; genetic interactions or specific phenotypes such as synthetic lethality between genes; gene regulation interactions between transcription factors and genes; and metabolic connections between enzymes and metabolites. In these networks, vertices represent genes, proteins, or other molecules, and edges represent specific interaction types [1,2].

An important current challenge is to develop methods to analyze these and other networks, such as social networks [3]. One challenge is to infer network structure by identifying subgroups of related vertices, which in the biological domain may be inferred to have similar functions. A second challenge is to predict links that might exist but which are not represented in the data. Missing links are prevalent in biological interactomes, where over half the true interactions may be absent from current data sets, and where spurious interactions may overwhelm true interactions in raw data [4]. Even most ambitious physical interaction mapping technique was limited to ~ 20% of the total possible interaction space [5]. Models based only on degree distribution have been unable to predict missing interactions [6].

Stochastic block models, in which vertices belong to groups and vertex-vertex interactions are determined by group membership, have shown promising results for network clustering in terms of probabilistic mixtures [7,8] (blocks) and admixtures [9] (blocks of blocks) of communities. Typically these models assume a flat structure of *K* top-level groups, which has the technical drawback of requiring a pre-specified value or a search over a pre-specifed range. A more serious problem, however, is a “resolution limit” in which the existence of large groups fundamentally prevents the discovery of small groups [10].

A recent hierarchical network model [11] proposed by Clauset, Moore, and Newman (CMN) provides a principled method for investigating structure at all levels by defining a probability distribution over network structures. This model avoids the resolution limit problem. It is also flexible in describing both assortative and disassortative networks. Unfortunately, it requires lengthy Markov chain Monte Carlo (MCMC) simulation to sample over network structures. More fundamentally, this model imposes an exhaustive hierarchical structure at both the top level (unrelated top-level groups are forced to merge together) and the bottom level (cohesive groups are exhaustively partitioned) of a network.

Here we describe a new algorithm, Hierarchical Agglomerative Clustering (HAC), that provides a fast, deterministic approximation for optimizing a network probability motivated by CMN. A key observation exploited by Newman and Leicht [12] is interactions with vertices outside a group often provide more information than within-group interactions. Methods that focus on within-cluster interactions, such as Bayesian Hierarchical Clustering [13], modularity scores [14], and even spectral methods [15] often miss this information. We use this information to drive accurate bottom-up clustering using a novel model selection strategy to identify groups to merge and to detect when a subtree should be collapsed into a single cluster, similar to Power Graph [16] but with a firm statistical foundation. A similar Bayesian model selection step determines when clustering should be terminated, yielding a set of top-level clusters lacking evidence for further hierarchical structure.

We then show that HAC achieves better accuracy in predicting missing links than other state-of-the-art algorithms. Moreover, the automated detection of structure at both the top and bottom level is shown to be expressive and flexible when applied to physical and genetic interactomes.

## Methods

### Preliminary definitions

#### Notation

A graph *G* is defined by a set of vertices *V* and edges *E* that connect pairs of vertices. This work considers undirected, unweighted edges with no self edges. Extensions to directed, weighted, and self-edges are possible but are not discussed here.

A “flat” *model.* A model ***M*** defines how vertices are collected into groups. These groups are denoted *C*_1_, *C*_2_, …, *C_K_* for a model with *K* groups. Each vertex is assigned to one of the *K* groups, and the groups are disjoint. This model can be summarized as ***M*** = {*C_k_* : *k ∈* 1, …, *K*}. Subscripts *u*, *v* typically refer to individual vertices, and subscripts *i*, *j*, *k* refer to groups.

Edge counts between groups can be summarized as  for *i* ≠ *j*, and  The binary variable *e_uv_ =* 1 for a *u* ~ *v* edge and 0 for the lack of an edge, or a hole. Total pair counts are defined as *t_ij_* = *n_i_n_j_* for *i* ≠ *j*, and *t_ii_= n_i_*(*n_i_* — 1)/2, where *n_i_* is the number of vertices within group *i*. Summary counts for holes are *h_ij_ = t_ij_* — *e_ij_.* For a given pair of groups *i* and *j*, the *e_ij_* edges are modeled as the result of *t_ij_* independent Bernoulli trials with parameter *θ_ij_*. The probability of the observed edges, conditioned on *θ_ij_*, is(1)

The maximum likelihood value  is obtained by setting *θ_ij_* to its maximum likelihood estimate with a uniform prior, . A fully Bayesian probability  is obtained by integrating out the nuisance parameter *θ_ij_*, again with a uniform prior:(2)

where Beta is the standard Beta function and *x^x^* = 1 for *x* = 0.

For a flat model, with *K*(*K* + 1)/2 parameters, the likelihood and fully Bayesian probability are(3)

#### Generalization to a hierarchical model

We can extend the notion of a model ***M*** to a hierarchical random graph (HRG) based on a model that successively merges pairs of groups [11]. This original model generates a binary dendrogram *T*. Each node *r* in this dendrogram represents the joining of graph vertices *L*(*r*) underneath the left sub-tree and vertices *R*(*r*) underneath the right sub-tree. With the same Bernoulli probability model (Eq.1) as a building block, *e_r_* and *h_r_* are defined as the total number of edges and holes crossing between the left and right sub-trees. We generalize this model for the case of multiple top-level nodes, which merge together into a flat structure using a full block model. We also generalize for tree structures that are not completely branching, yielding tree nodes that collect multiple graph vertices into a single group. Similar to Eq.3, letting ***M*** ≡ *T*, the likelihood ***L***(***M***) of a hierarchical model *T* and the corresponding probability *P*(***M***) of the graph given the model are(4)

Top-level terms  and  depend on the edges *e_rr′_* and holes *h_rr′_* crossing between the top-level groups *r* and *r′*, with *t_rr′_=e_rr′_+h_rr′_*. For all tree nodes,  and . For branching nodes (including the top-level nodes), the edges *e_r_* holes *h_r_* refer to those crossing between the left and right sub-trees; for non-branching terminals, *e_r_* and *h_r_* refer to the edges and holes for vertices within the terminal groups.

Sampling trees with MCMC provides excellent results for predicting missing links by accumulating  values for link probabilities between left and right sub-trees [11]. We have found that extending the MCMC approach to genome-scale networks is computationally burdensome. Approximation methods, such as a Variational Bayes approach [17], can reduce computational costs, but still require a good initial estimate of tree structure. Here we consider agglomerative approaches for finding trees *T* that optimize the objective function ***L***(***M***) and its fully Bayesian counterpart *P*(***M***).

### Agglomerative clustering

#### Maximum likelihood guide tree

Suppose currently there are *K* top-level clusters numbered 1 … *K* within the *R* total tree nodes. This model, ***M***, has *K*(*K —* 1)/2 + *R* total parameters. Merging two of the top-level nodes (and retaining the structure underneath each) gives a model with (*K—* 1)(*K—* 2)/2+ (*R+*1) parameters, a reduction of *K* — 2 parameters. Without loss of generality suppose we merge clusters 1 and 2 into a new cluster 1*′*, defining a new model ***M′***. The model likelihood ratio is(5)

There is a subtle but crucial difference between this agglomerative algorithm, which assumes a full block model for the top-level nodes, and the more standard approach with a star-like structure at the top with a single parameter governing the interactions between all pairs of top-level nodes. A starlike model with *K* top-level and *R* total nodes has *R*+1 parameters, and merging two groups increases the number of parameters by 1. The increase in parameters at each step, coupled with a maximum likelihood model, is liable to over-fit the group structure. A further problem is the model likelihood ratio for the star model,(6)

where  and similarly *h_b_* = *t_b_* - *e_b_* count the edges and holes between all pairs of top-level groups before merging 1 and 2, and *e*_12_ and *h*_12_ count the edges and holes just between groups 1 and 2. Under the star model, any two groups with the same values of *e*_12_ and *t*_12_ will have identical ratios . At the initial step, every pair of vertices will have one of two merging scores, depending on whether *e*_12_ = 1 or 0. Additional criteria are then required to avoid bad merges at the start of clustering. In contrast,  gathers information from shared patterns of connectivity with other grops. In particular, at the initial step when each group is a single vertex, , where the number of mismatches is 

#### Greedy agglomerative algorithm

The likelihood ratio  leads to an agglomerative algorithm that successively merges the two clusters have the largest value.

Initialize top-level clusters as {{*v*} : *v∈V*}

Initialize *K* ← *V*

**while***K* > 1 **do**

Find top-level clusters *i*,*j* with largest 

Add top-level cluster *r*; *L*(*r*) *= i* and *R*(*r*) *= j*

Remove clusters *i* and *j* from the top level

*K* ← *K* – 1

end while

We call this method **HAC-ML**. The time complexity of a naϊve implementation scales as *O*(*V*^4^), but using a priority queue, restricting possible merging pairs to clusters that share at least one common neighbor, and lazy evaluation of λ reduce the complexity to *O*(*EJ* log *V*), where *E* is the total number of edges and *J* is the average vertex degree.

#### Bayesian model selection for top-level and terminal clusters

A natural stopping criteria at the top level is obtained by augmenting , Eq. 5 with its fully Bayesian equivalent ,(7)

A reasonable stopping criterion is  for the best merge [18]. While there are *K*(*K* – 1)*/*2 possible merges, we do not include this factor in the stopping criterion.

Our previous work introduced a similar criterion for collapsing bottom-level clusters comparing a model with separate left and right sub-trees with a model all vertices collected in a single group [17]. Clusters with a single vertex are considered collapsed. During the merging process, if clusters 1 and 2 are selected for merging and are both collapsed, the probability ratio(8)

is calculated, where the subscripts indicate edges and holes within and between groups. The merged cluster is collapsed if . Clusters of two vertices are always merged because λ*^C^ =* 1. While there are  ways for the reverse process of splitting a cluster into two non-empty groups of sizes *n*_1_ and *n*_2_, we do not include this factor in the model selection.

#### Extension to multiple edge types

The HAC-ML algorithm is directly applicable to networks with multiple edge types. Rather than merging the edges into a single superimposed network, each edge type *α* defines its own likelihood ***L***^(^*^α^*^)^(***M***) and probability *P*^(^*^α^*^)^(***M***) for a particular model ***M*** . The full likelihood and full probability are then obtained as products over the edge types, ***L*** = ***∏****_α_****L***^(^*^α^*^)^ and *P* = ***∏****_α_ P*^(^*^α^*^)^.

### Performance Evaluation

#### Data preparation

Experimental evidence codes listed in BioGRID database (http://thebiogrid.org) provide a way to distinguish physical versus genetic interaction pairs. We built a physical network collecting all physically binding or interacting pairs and a genetic network restricted to negative interactions comprising to empirical evidence codes *Negative Genetic*, *Synthetic Growth Defect*, *Synthetic Haploin-sufficiency*, and *Synthetic Lethality.* We ignored redundant pairs within each type of network such that resulting graphs were undirected and unweighted. We then iteratively removed isolated or degree-1 vertices, as these provide scant information for clustering. For other non-BioGRID genetic interaction datasets we filtered out positively weighted pairs and applied the same iterative removal. In joint-network analysis, we restricted attention to the common intersection of genes.

#### Other methods

We compared HAC-ML with other deterministic methods: Fast Modularity (**CNM**; Clauset *et al.*[*14*]), Variational Bayes Modularity (**VBM**; Hofman and Wiggins [19], and Graph Diffusion Kernel (**GDK**; Qi *et al.*[*20*]). CNM is an efficient algorithm that directly optimizes Newman modularity [21]. VBM simplifies network data to one intra- and one inter-community probability distribution. For GDK by discriminating between even-length and odd-length paths, Qi *et al*. [*20*] improved link prediction performance, particularly for disassorative (bipartite-like) networks. We used the odd parity kernel with the recommended damping parameter set to 1.0.

#### Different merging scores

In addition, we also considered agglomerative clustering based on heuristic merging scores: (1) edge density, *ρ_e_;* (2) combined edge density and shared neighbor density, *ρ_e_* + *ρ_s_*; and (3) decomposed Newman modularity *Q* from CNM [21]. The edge and shared neighbor densities for merging clusters 1 and 2 are(9)(10)

The summations in *ρ_s_*(1, 2) runs over all vertices *u* not in groups 1 or 2, and the logical functions evaluate to 1 and 0. The Newman modularity for merging groups 1 and 2 is(11)

where *d_u_* and *d_v_* are vertex degrees and *E* is the total number of edges. This algorithm is essentially CNM, but retains the hierarchical structure defined by the merge order for link prediction (rather than predicting links based on the cut that maximizes modularity). Replacing  with *ρ_e_*, *ρ_e_* + *ρ_s_*, and *Q* yields algorithms **HAC-E, HAC-ES,** and **HAC-Q.**

#### Link prediction

We assessed correctness of a model in the framework of link prediction as presented in Henderson *et al*. [*8*]. Starting with a real-world network, training networks are generated by deleting a specified fraction of edges. A test set is defined by the held-out edges and a random choice of an equal number of holes. We then ran all methods on the training data set. The trained group structure provides maximum likelihood estimates for edges within and between clusters (Eq. 9). For VBM and CNM, we estimated edge densities between all pairs of clusters and within all clusters. For hierarchical models, we estimated densities between all left and right clusters at all tree levels. For GDK, each pair’s diffusion was directly used to rank pairs. Finally we assessed precision and recall of pairs in the test set ranked by link probability or GDK score. The counts of true positives (TP), false positives (FP), and false negatives (FN) as function of the number of predictions define the Precision, TP/(TP+FP), and the Recall, TP/(TP+FN). The F-score is the maximum value of harmonic mean of Precision and Recall. This test set definition is suitable for assessment, but overstates practical performance by reducing the number of negative test examples for a sparse network. Note that for large real-world networks, group assignments are generally unknown, making it difficult to assess group assignments directly.

#### Implementation

Algorithms were implemented in C++ and are available under an open source BSD license as supplementary material and from http://www.baderzone.org.

## Results and Discussion

### Data preparation

Interaction data was taken from BioGRID [22] (version 2.0.61) for physical interactions within *S.cerevisiae*, *A. thaliana*, *C. elegans*, *D. melanogaster*, and *H. sapiens*. Synthetic lethal and synthetic fitness defect genetic interactions were taken for *S. cerevisiae*. Additional genetic interaction data sets were collected from genome-wide Synthetic Gene Array (SGA) [23] and diploid-based Synthetic Lethality Analysis on Microarray (dSLAM) [24]. The largest network in this study contains roughly 5000 vertices and up to 100,000 interactions (Table 1).

**Table 1:** Network data sets

Name	*V*	*E*		Kind	Organism	Source
Arabidopsis	777	1,831	4.71	Physical	*A. Thaliana*	BioGRID^1^
Celegans	1,089	2,842	5.22	Physical	*C. elegans*	BioGRID^1^
Drosophila	4,692	19,876	8.47	Physical	*D. melanogaster*	BioGRID^1^
Human	6,094	26,112	8.57	Physical	*H. sapiens*	BioGRID^1^
Yeast-PPI	5,105	50,542	19.80	Physical	*S. cerevisiae*	BioGRID^1^
Yeast-GEN	4,763	85,855	36.05	Genetic	*S. cerevisiae*	BioGRID^1,2^
SGA	4,398	108,369	49.38	Genetic	*S. cerevisiae*	Costanzo *et al*.^3^
dSLAM	627	4,710	15.02	Genetic	*S. cerevisiae*	Pan *et al*.^4^

*Symbols*: *V*, number of vertices (genes/proteins); *E*, number of edges (interactions);  , average degree. *Data sources*: (1) BioGRID 2.0.61 [22]; (2) We selectively included “Negative Genetic”, “Synthetic Growth Defect”, “Synthetic Haploinsufficiency”, “Synthetic Lethality” experiments; (3) Supp. Data S4, intermediate cutoff, of Costanzo *et al*. [23]; (4) Supp. Table S1 of Pan *et al*. [24].

### Empirical evaluation

Summary results for link prediction demonstrate overall superior performance by HAC-ML (Table 2). Of the 8 real-world networks, HAC-ML is top or tied for top in link prediction 6 times, followed by GDK for 2, CNM for 2, and VBM for 1. These summary results are for 7.5% of known edges held out, and supplemented with an equivalent number of holes selected at random as an 85/15 cross-validation set.

**Table 2:** Link prediction performance of 85/15 cross validation (7.5% of observed edges held out).

Physical interactions							
Data	HAC-ML	GDK	CNM	VBM	HAC-ES	HAC-E	HAC-Q

Yeast-PPI	**0.79**±0.5	0.69±0.3	0.69±0.7	0.76±0.4	0.71±0.5	0.69±0.7	0.69±0.8
Drosophila	**0.73**±0.8	0.66±0.2	0.67±0.4	0.70±0.4	0.67±0.3	0.67±0.3	0.67±0.4
Human	0.73±0.9	**0.75**±0.7	0.71±0.5	0.70±0.6	0.67±0.4	0.68±0.5	0.69±1.0
Celegans	**0.68**±1.5	0.67±1.3	**0.68**±1.3	0.66±0.6	0.66±0.8	0.66±0.7	0.67±0.8
Arabidopsis	0.80±8.3	**0.92**±2.2	**0.92**±3.2	0.90±3.6	0.78±11.0	0.87±10.8	0.88±11.4
Genetic interactions							
Data	HAC-ML	GDK	CNM	VBM	HAC-ES	HAC-E	HAC-Q

Yeast-GEN	**0.78**±2.3	0.67±0.0	0.69±0.7	0.74±6.0	0.73±0.8	0.67±0.1	0.69±0.7
SGA	**0.76**±1.5	0.67±0.0	0.67±0.2	**0.76**±0.3	0.70±0.2	0.67±0.0	0.69±0.2
SLAM	**0.92**±1.0	0.91±0.5	0.68±0.8	0.67±0.3	0.84±2.9	0.76±1.0	0.67±0.3

First numbers indicate an average *F*_1_ score of multiple experiments and second numbers following ± sign are standard deviations of last-digit (multiplied by 100).

**Figure 1:**
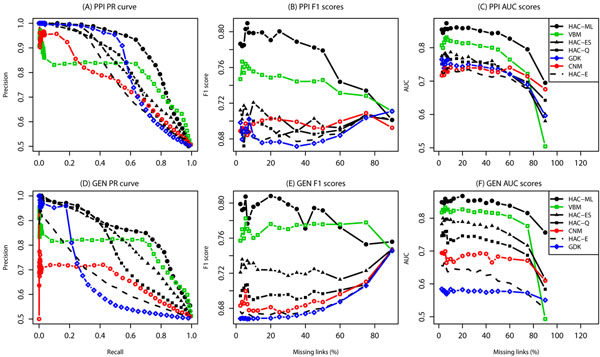
**Link prediction performance of Yeast data sets. ***A*: Precision Recall (PR) curve of 80/20 cross-validation experiment (CV) in YEAST-PPI dataset (10% missing links); *B*: F1 scores over different fractions of missing links in YEAST-PPI dataset from 1.5% to 90%; *C*: Area under ROC curve (AUC) scores over different fractions of missing links in YEAST-PPI dataset; *D*: PR curve of a 80/20 CV in YEAST-GEN dataset; *E*: F1 scores in YEAST-GEN dataset; *F*: AUC scores in YEAST-GEN dataset.

More detailed results are provided for two of the largest networks, Yeast-PPI physical interactions (Fig. 1A,B,C) and Yeast-GEN genetic interactions (Fig. 1D,E,F). The HAC-ML method dominates along the precision-recall curve, and also generally performs best over many fractions of left-out edges (Fig. 1B,C,E,F). The high-precision region of the HAC-ML prediction generally extends further than the other methods (Fig. 1A,D).

Among top-ranked pairs, the flat models CNM and VBM perform worse than the hierarchical models. The performance of CNM is improved to nearly the performance of HAC-ML by using HAC-Q to determine the merge order. The poor performance of CNM and VBM in the high-precision region may reflect the inherent resolution limit of a flat model [10] that hierarchical models do not appear to be limited.

Methods that consider shared neighbors, including HAC-ML and GDK, also perform better than methods that ignore this information, such as HAC-E. Shared neighbors are strong predictors of missing links in networks of protein interactions [25] and genetic interactions [26]. Methods that consider shared neighbors, as opposed to just modularity or density, perform better for disassortative networks such as Yeast-GEN. The VBM method, which assumes homogeneous groups, may also work incorrectly when applied to networks with a mix of assortative and disassortative group structures.

### Multi-resolution views of a physical interaction network

**Figure 2:**
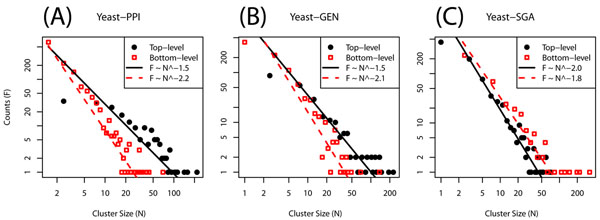
**Cluster size distribution.*** Black closed circles*: Counts of top-level clusters; *Black solid line*: Maximum likelihood power-law fit; *Red open squares*: Counts of low-level clusters; *Red dashed line*: Maximum likelihood power-law fit; *A, B, C*: Each panel respectively corresponds to the result of YEAST-PPI, YEAST-GEN, and YEAST-SGA datasets.

Bayesian model selection provides criteria for collapsing homogenous bottom-level clusters and for identifying top-level clusters that should not be merged. The size distributions for top-level and bottom-level clusters have long tailed distributions (Fig. 2). Power-law fits for maximum likelihood [27] yield exponents close to 2, albeit over only a decade of sizes.

**Figure 3:**
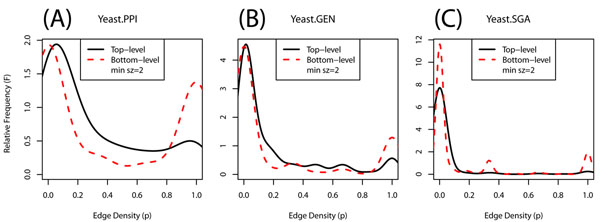
**Interaction enrichment within clusters. ***Black solid lines*: Edge-density distribution of the top-level clusters; *Red dashed lines*: Edge-density distribution of the bottom-level clusters. *A, B, C*: Each panel respectively corresponds to the result of YEAST-PPI, YEAST-GEN, and YEAST-SGA datasets.

Edge densities within top-level clusters and bottom-level clusters have bimodal distributions, including edge densities of both 0 and 1 (Fig. 3). Clusters with density 0 can be generated when unconnected vertices share one or more interaction partners, a frequent pattern in both physical and genetic interaction networks. Standard algorithms for identifying densely connected subnetworks [1,2,28] perform poorly in these cases, whereas algorithms based on shared neighbors can still perform well [29].

**Figure 4:**
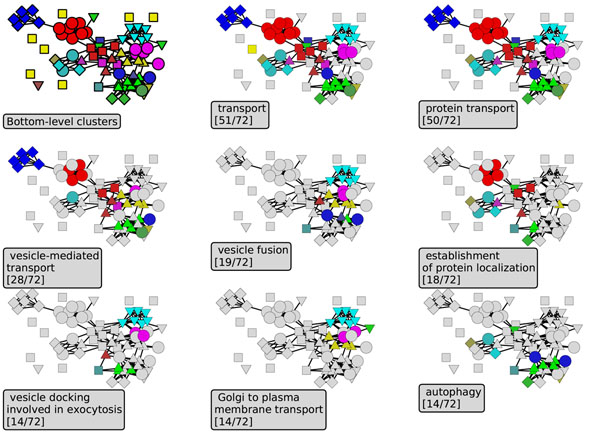
**Protein transport complex. ***Bottom level clusters*: Different shapes and colors in the topmost and leftmost panel indicate different bottom-level clusters; *Other panels*: Each box indicates one GO keyword and its enrichment within the subnetwork, and vertices belonging to this GO category are highlighted by non-gray colors.

A representative example of a top-level cluster with bottom-level structure is the protein transport complex discovered in the Yeast-PPI network (Fig. 4). This cluster, with 72 vertices, has a hierarchical structure with 4 layers branching down to over 10 bottom-level clusters. The bottom-level clusters include examples both of cliques (fully connected sets of vertices) and proteins that do not interact with each other but share common neighbors, including neighbors in other top-level groups.

Visual inspection indicates that the bottom-level clusters are subsets of known GO annotation categories, and may provide greater resolution than existing bottom-level GO categories. These results also indicate connections between GO categories learned from high-throughput data. An example is process of autophagy, which starts by forming a membrane-bound component that engulfs excess cytosolic proteins and make degraded in lysosome or other vacuoles [30,31]. Therefore “vecicle fusion” and “vesicle-mediated transport” are its mechanistic processes; a proper “protein localization” and targeting is required. Connections with plasma membrane proteins have become recently known, suggesting that plasma membrane is the source of autophagosome and autophagy is initiated by *de novo* assembly of proteins and lipids [32,33]. As autophagy is a response to starvation [30] to re-use available intracellular resources. We find that disjoint low-level clusters correspond to “autophagy” and “golgi to plasma membrane transport”, suggesting that different proteins are responsible for transport in each direction. Moreover seemingly distant relationship to “exocytosis” is under investigation [34].

### Synergy in mixed networks

The extension to multiple edge types was used to compare link prediction for single yeast networks to link prediction from simultaneous analysis of physical and genetic interaction data (Table 3). Little evidence for synergy is apparent: predictions for a specific network are not improved by adding data from a second or third network. This behavior has been observed before for joint analysis of physical and genetic interactions [20,35].

**Table 3:** Link prediction performance of joint analysis.

HAC-ML	Prediction of		
Trained by	PPI	SGA	GEN
PPI	**0.75**±1.6		
SGA		**0.77**±1.0	
GEN			0.78±1.4
PPI+SGA	0.69±0.5	0.73±0.8	
PPI+GEN	0.71±1.1		**0.79**±0.5
SGA+GEN		**0.77**±1.0	0.78±1.1
PPI+SGA+GEN	0.68±1.2	0.73±0.3	0.78±0.6

Evaluation scheme was 85/15 cross-validation. First numbers indicate an average *F*_1_ score of multiple experiments and second numbers following ± sign are standard deviations of last-digit (multiplied by 100).

This lack of synergy may arise from high-throughput studies exploring different subsets of genes and proteins. Moreover our joint analysis assumes different types of edges are generated under a common group structure, but this pattern might be disrupted by a large fraction of false positive interactions, or some edge types might conflict with others. In presence of prevalent false positive interactions, physical and genetic interactions might not be *directly* complementary or orthogonal to each other in contrary to Kelley *et al*. [36]. In our simulation study, where orthogonality is well-preserved, HAC-ML trained by multiple data sources significantly outperformed (results not shown). To resolve this issue, a kernel-based method used by the previous studies [35] can be beneficial, but this is an open research problem.

## Conclusions

The hierarchical agglomerative clustering methods HAC-ML is effective at discovering structure in real-world networks, with the ability to resolve both top-level and bottom-level groups. It provides superior performance for link prediction when applied to real-world networks, with a good tradeoff between efficiency and accuracy.

A general weakness of deterministic optimization heuristics is the possibly of becoming trapped in a local minimum. A more fundamental weakness is that different aspects of cross-cutting network structure may be reflected by multiple pertinent local minima. Even so, the group structure generated by HAC-ML can be used as a starting point for MCMC sampling over tree structures, which can provide better results than any single tree [11].

Unlike many agglomerative algorithms which effectively introduce a new parameter every time two groups are merged, HAC-ML starts from a full model and removes parameters at each step. This approach gathers information from shared interaction patterns in building a guide tree, and then uses Bayesian model selection to collapse the bottom level of the tree and terminate the clustering at the top level. Extensions to joint analysis of multiple networks are provided, and extensions to more complex networks with weighted, directed, and time-varying edges are easily envisioned within the same probabilistic framework.

## Competing interests

The authors declare that they have no competing interests.

## Authors' contributions

YP and JSB developed the methods, analyzed the results, and wrote the manuscript. YP implemented the methods and performed the calculations.
